# Chemical and Bioassay Techniques to Authenticate Quality of the Anti-Leishmanial Drug Miltefosine

**DOI:** 10.4269/ajtmh.14-0586

**Published:** 2015-06-03

**Authors:** Harparkash Kaur, Karin Seifert, Geoffrey E. Hawkes, Gregory S. Coumbarides, Jorge Alvar, Simon L. Croft

**Affiliations:** London School of Hygiene & Tropical Medicine, London, United Kingdom; School of Biological and Chemical Sciences, Queen Mary, University of London, London, United Kingdom; Drugs for Neglected Diseases initiative (DNDi), Geneva, Switzerland

## Abstract

Miltefosine, an effective oral treatment of visceral leishmaniasis (VL), was selected in May 2005, by the governments of India, Nepal, and Bangladesh for the elimination of VL. However, abnormally high treatment failure rates reported in patients in Bangladesh, given a miltefosine generic product (“Miltefos”, Popular Pharmaceuticals Ltd.) during 2008, led the World Health Organization (WHO) to procure this formulation for quality testing. Proton (^1^H) and phosphorous (^31^P) nuclear magnetic resonance (NMR) analyses of the Miltefos™ capsules did not give the peaks defined for Impavido^®^, the quality assured VL treatment product from Aeterna Zentaris. Contents of capsules of Impavido^®^ yielded expected peaks for miltefosine (m/z 408.33 for the protonated parent ion and m/z 183.99 plus *m/z* 124.8 the fragment ions) that were absent in the Miltefos™ capsules. Furthermore, testing using an in vitro *Leishmania donovani* intracellular amastigote—macrophage model, yielded EC_50_ values of between 2.55 and 4.06 μg/mL and 3.02 to 5.92 μg/mL for extracts from the Impavido^®^ capsules and the miltefosine standard, respectively. Lack of significant anti-leishmanial activity of Miltefos™ capsules was identified in this assay even at concentrations up to 100 μg/mL. Capsules of Miltefos™ were classified as falsified (absence of stated active pharmaceutical ingredient) by three methods—NMR and mass spectrometry analysis and bioassay.

## Introduction

The leishmaniases are a group of neglected tropical diseases caused by parasites of the genus *Leishmania*. The main disease manifestations are cutaneous leishmaniasis (CL) and visceral leishmaniasis (VL). Active VL has a high fatality rate in almost all cases and requires treatment with anti-leishmanial drugs. Recent estimates for VL suggest between 200,000 and 400,000 cases and 20,000 and 40,000 deaths per year worldwide with over 90% of cases occurring in India, Bangladesh, Sudan, South Sudan, Brazil, and Ethiopia.^1^ Current drugs for VL include pentavalent antimonials, amphotericin B (formulated as salt with deoxycholate in Fungizone^®^ or in a liposomal form as AmBisome^®^), miltefosine, and paromomycin. Multi-drug therapy by co-administration regimes is also being pursued as an improved treatment strategy for VL.^2^

Miltefosine, originally developed as an anticancer drug, is the first effective oral treatment of VL and CL. It was determined to be safe and effective for the treatment of Indian VL following a trial, in which 282 of 299 (94%) patients were cured with an oral dose of 2.5 mg/kg of miltefosine given daily for 28 days as monotherapy.^3^ It was then registered in India for oral treatment of VL in March 2002.^4^ The drug is well-tolerated, except for mild gastrointestinal side effects, but its teratogenic potential hampers its general use in the clinic, particularly for women of childbearing age. It has a long elimination half-life and accumulates in the body during treatment.^5^

Miltefosine (currently available through Paladin Laboratories, Inc., Montreal, Canada) under the trade name Impavido^®^, was adopted by the governments of India, Nepal, and Bangladesh as an oral treatment of VL in the program to eliminate this disease by 2015.^6^ As part of the elimination program, and to improve procurement of this necessary intervention, the Bangladesh authorities identified a local company (Popular Pharmaceuticals Ltd., Dhaka, Bangladesh) that manufactured generic miltefosine, “Miltefos”, for supply.

“Miltefos” was introduced as a first-line therapy for VL in Bangladesh in 2008. Bangladesh authorities and World Health Organization (WHO) were reporting unexpected levels of treatment failures shortly after its introduction in 2008, a different clinical response to that determined in India and Nepal.^4^,^7^ Both “Miltefos” and Impavido^®^ were administered simultaneously to patients and it became immediately apparent that the group on “Miltefos” did not show the anticipated treatment response. As part of the investigation to establish the basis for this difference, samples of “Miltefos” were sent to the Academic Medical Center, University of Amsterdam and to the London School of Hygiene and Tropical Medicine (by WHO). The group in Amsterdam characterized representative drug samples from batches labeled 10 mg miltefosine and 50 mg miltefosine using a liquid chromatography–tandem mass spectrometry (MS/MS) methodology developed with a sensitivity as low as 4 ng/mL in human plasma.^8^,^9^ Further studies using second drug samples and examination of blood samples from five patients, confirmed that 1) no active pharmaceutical ingredient could be identified in the “Miltefos” batches and, 2) miltefosine could not be detected in the blood of the patients who had been administered “Miltefos” capsules.^10^

We describe here the results of studies performed on Miltefos™ batches received by WHO in 2008 at the same time as those sent to the group in Amsterdam. Our data, acquired using nuclear magnetic resonance (NMR) spectroscopy, mass spectrometry (MS), and bioassay analyses, supports the findings of Dorlo and others^8^,^9^ and further characterizes this falsified drug by complementary methodologies.

## Materials and Methods

### Samples.

The WHO received in 2008, requests from their South East Asia Regional Office (SEARO), Delhi, and the Directorate General of Health Services of Bangladesh to test a generic miltefosine formulation produced by Popular Pharmaceuticals Ltd., for quality issues. The WHO SEARO sampled a number of blister packs randomly from warehouses but without a precise methodology. Three sealed packs were collected from the subdistrict health centers (upazila health level) where the drugs were supplied by the program for patient treatment. The samples were collected jointly by the Representatives of Directorate of Health, WHO-Bangladesh, Popular laboratories, and Directorate of Drug Administration and sent to WHO-Geneva by the Director General of Health Services. WHO-Geneva coordinated the work with the London School of Hygiene and Tropical Medicine (LSHTM) and Bangladesh authorities. Miltefos™ capsules were received from WHO SEARO and the WHO Department of Neglected Tropical Diseases and Department of Essential Medicines and Pharmaceutical Policies, Geneva.

Impavido^®^ capsules (containing 50 mg and 10 mg miltefosine, batch nos. 8J7717 and 8H7859, respectively) and the active pharmaceutical ingredient (API) miltefosine (D-18506, batch no. 986356) were a gift from Paladin Laboratories Inc. (Montreal, Canada). Upon arrival capsules and API were stored at an air conditioned laboratory (21°C).

### Chemicals.

Methanol (99.8 + % (GLC) and water were from Thermo Fisher Scientific, Hemel Hempstead, UK. Deuterated chloroform the solvent used for NMR spectroscopy and tetramethylsilane the chemical shift reference were purchased from Sigma-Aldridge, Dorset, UK. Both high performance liquid chromatography grade methanol and water of purity grade were from Thermo Fisher Scientific. Giemsa's stain (improved R66 solution Gurr) was purchased from VWR, East Grinstead, West Sussex, UK, RPMI-1640 medium from Sigma, UK, and fetal calf serum (FCS) from Harlan-Sera Laboratory, Leicestershire, UK.

### ^1^H and ^31^P NMR spectroscopy analysis.

Proton and phosphorous (^1^H and ^31^P) are high-resolution atomic nuclei, both having spin quantum number I = 1/2. An advantage of ^1^H NMR is that it is more sensitive than ^31^P by a factor about 10. However, ^31^P has a greater chemical shift range than ^1^H. In addition, a phosphorous-containing molecule typically has considerably more chemically distinct proton environments than it has phosphorous environments. Therefore, ^1^H NMR spectra from such molecules are inherently considerably more complex than ^31^P spectra, but at the same time the ^1^H spectra have a far greater overall information content. In this case, as the molecule of miltefosine has a phosphorous atom it was judged to be appropriate to analyze using ^31^P NMR.

Miltefosine (API reference compound) and the contents of the capsules were dissolved in deuterated chloroform (CDCl_3_), which dissolves the API but not the excipients. The solute filtered through ordinary tissue paper and transferred into NMR tubes followed with the addition of the chemical shift reference (TMS; tetramethylsilane). The chemical shift reference for ^31^P spectra was external 85% H_3_PO_4_. The tube was then inserted into the instrument—Bruker Avance 400 NMR spectrometer (Coventry, UK) measuring the proton (^1^H) and phosphorous (^31^P) spectra at 400 and 162 MHz, respectively.

### Extraction of capsule content and preparation of stock solutions for MS and bioassay analysis.

Capsules of Miltefos™ and Impavido^®^ were opened and the contents emptied into pre-weighed glass vials. The weight of capsule contents was recorded and 5 mL of a 1:1 (v/v) mixture of methanol and water added. Vials were briefly shaken and placed in a cooled ultrasound water bath for 30 minutes with 2–3 times vortexing in between. Vials were left to stand in the air conditioned laboratory (21°C) for 30 minutes after which 2 mL of solutions were transferred to Eppendorf tubes. Tubes were centrifuged at 13,000 rpm for 10 minutes (SANYO; Micro Centaur; MSE, London, UK) and the supernatant transferred to new Eppendorf tubes. One aliquot was filter sterilized by passing the solution through a Minisart^®^ syringe end filter (pore size 0.2 μm) and used as stock solution for the bioassay. The other aliquot was used in liquid chromatography mass spectrometry analysis. The API stock was prepared in the same solvent as capsule extracts.

### MS analysis and data processing.

Mass spectrometry full scan and MS/MS analyses were performed on the Thermo Finnigan LCQ ion trap instrument with an electron spray ionization (ESI) source (THermoFinnigan, Hemel Hempstead, UK). The ESI source was operated in the positive ion mode with the following operating conditions optimized for miltefosine by infusing a solution (2 mg/mL reference standard in methanol/water; v/v; 1:1) using the syringe pump at a flow rate of 2.5 μL/min; transfer capillary temperature 220°C, spray voltage 4.55 kV, capillary voltage 3.29 V, and sheath gas flow 30 (arbitrary units). Nitrogen was used as the nebulizing gas (by Peak, Scientific, Inchinnan, Scotland, UK; generator). Data acquisition and processing was performed using Xcalibur software (version 2.0.7 SP1). The cumulative scans were recorded over a mass range of *m/z* 110–430 for 3 minutes to be sure that all the ions in the sample were included in the spectrum. Solutions were directly infused to the system using the syringe pump. The probe was washed between each sample analysis first with water and then with methanol to ensure that there was no carryover of the previous sample.

### Drug activity evaluation.

The anti-leishmanial activity of capsule extracts and API was evaluated in a standard assay as described previously.^11^ Briefly, mouse peritoneal macrophages (PEMs) were harvested from CD1 mice (Charles River, Margate, UK) by lavage 24 hours after intraperitoneal injection of 2% soluble starch in PBS (both from Sigma). Macrophages were plated in Lab-tek 16-well chamber slides (VWR) at a density of 5 × 10^4^ cells/well in RPMI 1640 medium plus 10% hi-FCS. Macrophages were left to adhere overnight at 37°C, 5% CO_2_, and infected with freshly harvested *Leishmania donovani* amastigotes (MHOM/ET/67/HU3 in experiment 1 and MHOM/IN/80/DD8 in experiment 2) at an infection ratio of 1 macrophage: 5 amastigotes. After incubating cultures overnight at 37°C, 5% CO_2_ non-phagocytosed amastigotes were gently washed off and miltefosine added at the final concentrations of 100, 20, 4, and 0.8 μg/mL. A 4-point dilution series spanning the concentration ranges used here was based on knowledge of miltefosine activity and toxicity towards host cells. Each concentration was tested in quadruplicate. Controls received serum substituted medium only or serum substituted medium containing 0.5%, 0.1%, and 0.02% (v/v) methanol. Infected macrophages were incubated with drug dilutions for a total of 3 days at 37°C, 5% CO_2_.

At assay endpoint, slides were fixed with 100% methanol and stained with 10% Giemsa in water. The average of the quadruplicates of cultures receiving medium only was taken to serve as 100% control against which the percentage inhibition of infected macrophages in treated cultures was calculated. Effective concentrations at the 50% level (EC_50_ values) were estimated with the non-linear sigmoidal curve-fitting Levenburg Marquardt algorithm (Microsoft *xl*fit, ID Business Solution, Guildford, Surrey, UK). Dose responses were additionally checked manually from the raw data (percentage infected macrophages/inhibition) to confirm fitness of data. Toxicity against macrophages was noted when no macrophages were present in a well or macrophage morphology and/or number clearly changed when compared with untreated controls. All experiments involving animals were conducted under license in accordance with UK Home Office License no. PPL5937.

## Results

### Proton nuclear magnetic resonance spectroscopic ^1^H NMR spectra.

**Figure 1.**
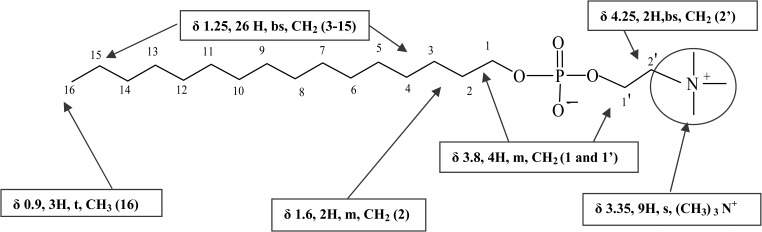
Chemical structure with the numbering scheme of atoms of the miltefosine molecule used in the description of the nuclear magnetic resonance (NMR) spectral peaks plus the chemical shift (δ) for the protons and their position on the molecule.

**Figure 2.**
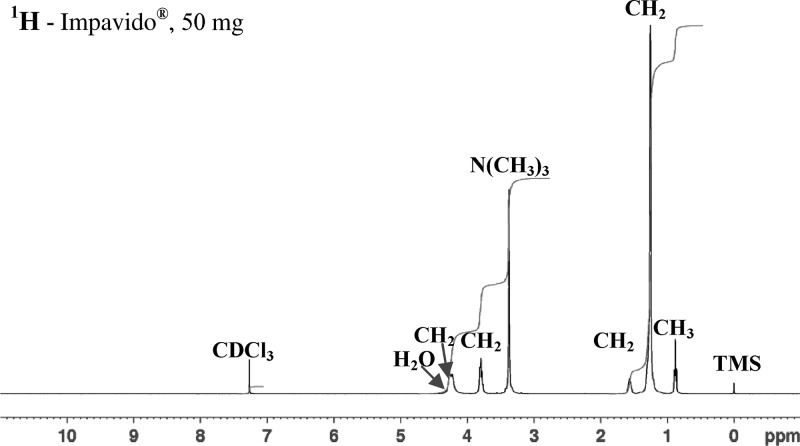
The ^1^H NMR (CDCl_3_, 400 MHz) spectra of an extract of Impavido^®^, 50 mg miltefosine capsules show position of the peaks and their assignment to the protons in the structure of miltefosine (see Figure 1) δ 0.90, 3H, t, CH_3_(16), δ 1.25, 26 H, bs, CH_2_(3–15), δ 1.60, 2H, m, CH_2_(2), δ 3.35, 9H, s, (CH_3_) _3_ N^+^, δ 3.80, 4H, m, CH_2_(1 and 1′), δ 4.25, 2H,b s, CH_2_(2′).

Miltefosine (hexadecylphosphocholine, Figure 1

, shows the numbering of the scheme of atoms of the molecule used to describe the NMR spectral peaks) reference standard was dissolved completely in CDCl_3_ to give a clear solution and the spectral peaks, which matched those resulting from the extracts of Impavido^®^ (50 and 10 mg miltefosine) as shown on Figure 2

**Figure 3.**
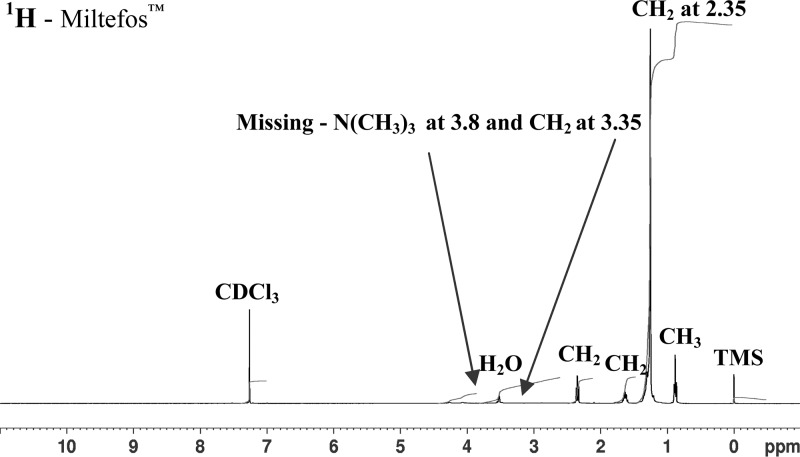
The ^1^H NMR (CDCl_3_, 400 MHz) extract of Miltefos™, 50 mg miltefosine capsules from Popular Pharmaceuticals Ltd.) show the absence of the peaks expected if miltefosine (see Figure 2, CH2(2′) at 4.25, CH2(1 + 1′) at 3.8, N(CH3)3 at 3.35) was present. Shows the presence of a new peak for CH_2_ at 2.35.

**Figure 4.**
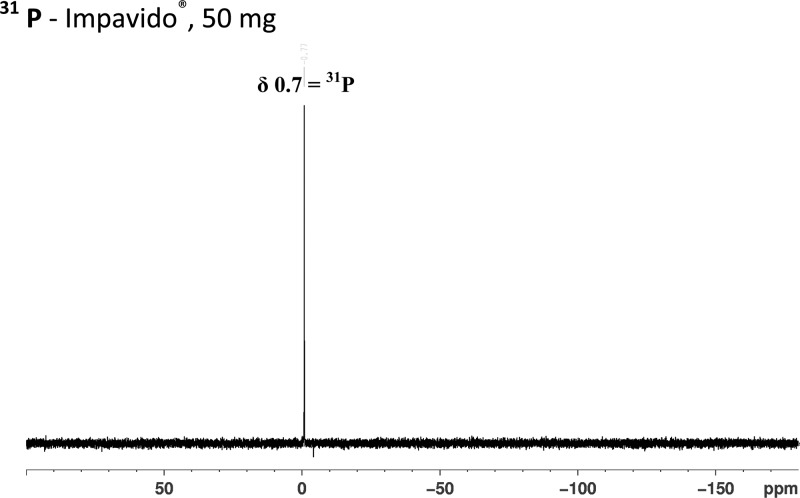
The ^31^P NMR (CDCl_3_, 162 MHz) spectra of an extract of Impavido^®^, 50 mg miltefosine capsules show the presence of the singlet peak for phosphorous at δ 0.7.

**Figure 5.**
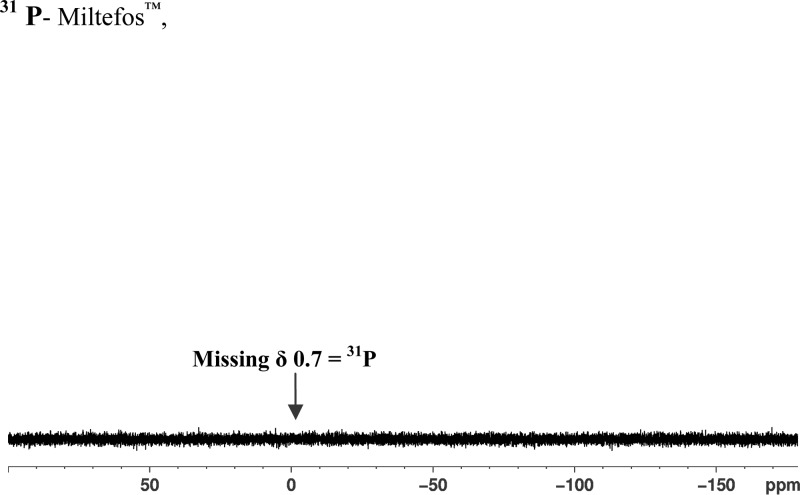
The ^31^P NMR (CDCl_3_, 162 MHz) extract of Miltefos™, 50 mg miltefosine capsules from Popular Laboratories. The expected singlet peak for phosphorous at δ 0.7 is not present.

. The ^1^H NMR spectrum of the Miltefos™ capsules (Figure 3

) does not show any signals from a phosphocholine group, viz CH_2_(1′) at δ 3.8, CH_2_(2′) at δ 4.25, N(CH_3_)_3_ at δ 3.35. In addition, there is no signal at δ 3.8 due to CH_2_(1) of the hexadecyl chain. Instead, a new signal is seen at δ 2.35 (integration for CH_2_). The presence of the phosphocholine group in the is seen in the ^31^P NMR spectrum of the Impavido^®^ capsules Figure 4

but is lacking in the spectrum from the Miltefos™ capsules (Figure 5).

**Figure 8.**
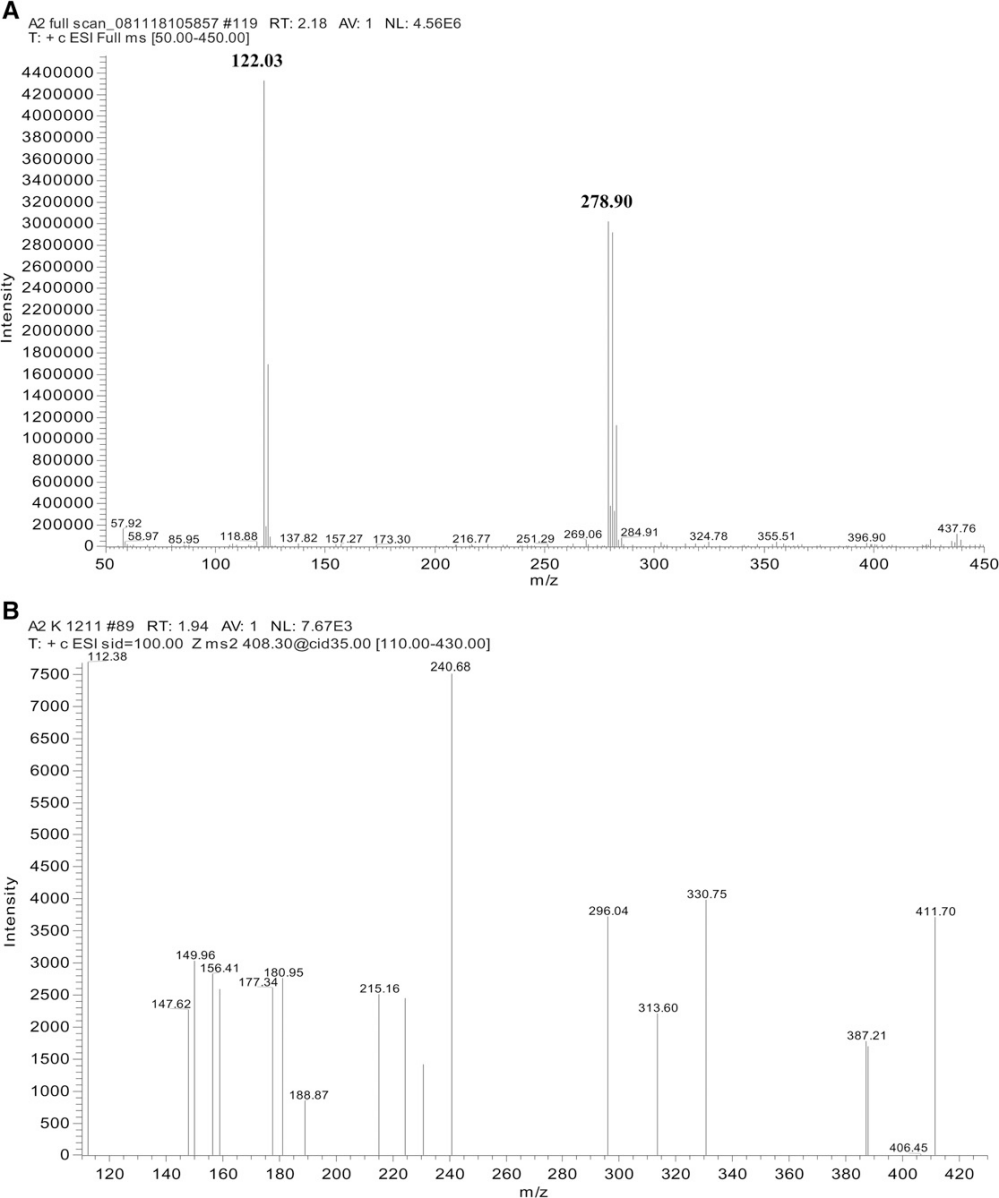
(**A**) The full scan mass spectrum of Miltefos™, 50 mg miltefosine capsules from Popular Laboratories does not show peak at m*/z* 408.33 for the active ingredient and the origins for the peaks at 122.03 and 278.90 are unknown. Intensity of the cumulative signals over 3 minutes is 4.56 × 10^6^. (**B**) The tandem mass spectrometry (MS/MS) product ion scan of Miltefos™, 50 mg miltefosine capsules from Popular Laboratories does not show peak at m*/z* 408.33 for the active ingredient and the product ions at *m/z* 183.99 and *m/z* 124.8.

It is of interest to determine the nature of the compound(s) detected in the ^1^H NMR spectrum of the Miltefos™ capsules. The spectrum in Figure 3 shows the signals expected of a hexadecyl derivative C_16_H_33_X, with the signal of the terminal CH_2_ group (–CH_2_–X) at δ 2.35. There are no other significant ^1^H NMR signals to be considered. Inspection of ^1^H NMR databases† ‡ indicates that –X is unlikely to be an oxygen substituent or halogen because the –CH_2_X ^1^H NMR signal (δ 2.35) is too low in frequency. The exact nature of X is not certain, as there is no information available on the procedures followed for the production of Miltefos™ capsules. Candidate structures for the substituent X, based upon the position of the CH_2_X ^1^H NMR signal, are 1) X = SH (−CH_2_SH at‡ δ 2.56 in hexadecyl mercaptan) and 2) X = (C=O) C_16_H_33_ (–CH_2_CO– at† δ 2.4 in similar di-*n*-alkylketones). However, a very good match occurs for X = CO_2_H; –CH_2_CO_2_H at δ 2.35–2.36 in hexadecanoic acid (palmitic acid) C_15_H_31_CO_2_H and heptadecanoic acid§ C_16_H_33_CO_2_H both of which occur naturally in milk and certain meat fats. Palmitic acid has a molecular weight of 256.42 and a peak is seen on the MS at 278.90 (Figure 8A), which may be thus assigned to ([M + Na]^+^). †Human Metabolome Database www.hmdb.ca.
‡www.molbase.com.
§Normal errors, ±5%, on integration of ^1^H NMR signals accommodates either structure.

### MS (full scan and MS/MS) analysis.

**Figure 6.**
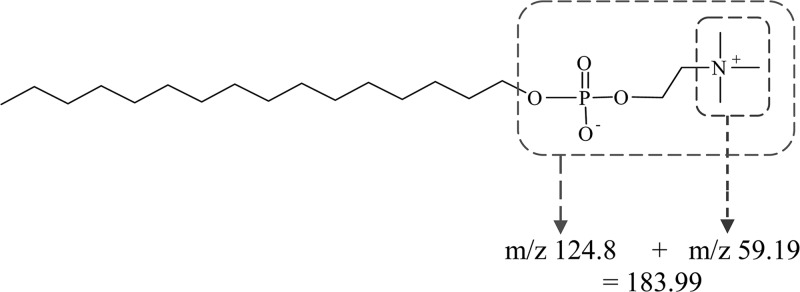
The chemical structure of miltefosine and the m/z fragmentation resulting in the daughter ions (183.99, 124.8, and 59.19) resulting from the protonated mass ion [M + H]^+^ at m/z 430.

The proposed fragmentation pattern of the protonated ion of miltefosine is depicted in Figure 6

**Figure 7.**
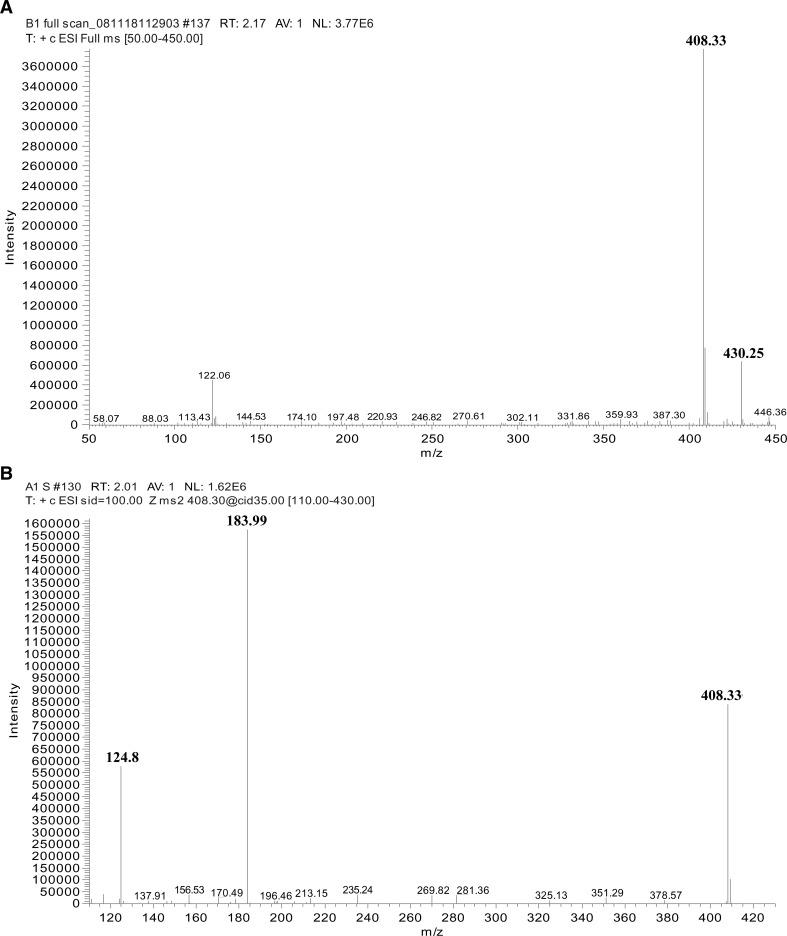
(**A**) The full scan mass spectrum of Impavido^®^, 50 mg miltefosine capsules and the peaks caused by the presence of the active ingredient at *m/z* 408.33 and the sodium salt *m/z* 430.25. Intensity of the cumulative signals over 3 minutes is 3.77 × 10^6^. (**B**) Showing the tandem mass spectrometry (MS/MS) product ion scan of Impavido^®^, 50 mg miltefosine capsules, and the peaks caused by the presence of the active ingredient at *m/z* 408.33 and the product ions at *m/z* 183.99 and *m/z* 124.8.

. A full scan (110 – 450 *m/z*) spectrum will detect the presence or absence of miltefosine in the capsules by checking for the anticipated protonated parent ion [M + H]^+^ at *m/z* 408.33 and the sodium adducts [M + Na]^+^ at *m/z* 430.25 in the solutions (Figure 7A

). (The sodium ion Na^+^ detected in the ESI trap is postulated to originate from the solvents and/or glass ware used). The protonated parent ion was then induced to fragment in the collision cell. The MS/MS fragment ion spectrum will result in the m*/z* 183.99 fragment that corresponds to the loss of the C_16_− alkyl chain and further cleavage of this precursor fragment ion results in the product ion at *m/z* 124.8 (Figure 7B).

Dilutions of the reference standard (2 mg/mL) were carried out using the solvent (methanol: water; v/v; 1:1) down to 0.1 μg/mL and infused into the probe using the syringe pump to determine the limit of detection of our method.

Direct mass spectral analysis was used to detect the presence or absence of the compound – miltefosine, the active ingredient as stated on the labels of generic miltefosine capsules from Impavido^®^ (Figure 8A and B) and Miltefos™ (Figure 8A and B). The presence of the protonated parent ion peak at m*/z* 408.33 on the full-scan analyses and of m*/z* 183.99 and *m/z* 124.8 on the MS/MS product ion scan were used to confirm the presence or absence of the active ingredient – miltefosine in the capsules. Summary results of the NMR and MS analyses of the capsules of Miltefos™ and Impavido^®^ are shown in Table 1.

### Anti-leishmanial activity of extracted capsule contents and API.

Drug assays were validated through the use of reference standards (API prepared in two different stock concentrations corresponding to the stocks theoretically obtained from capsule extracts), confirmed to give EC_50_ values in agreement with known anti-leishmanial activity of miltefosine.^11^ Dose response curves were obtained for extracts from Impavido^®^ capsules labeled to contain 50 and 10 mg miltefosine, which allowed the estimation of EC_50_ values. These were consistent with values obtained from the reference standard and within the expected range of known miltefosine activity in vitro. Values at the EC_50_ level ranged from 2.68 μg/mL to 4.02 μg/mL (corresponding to 6.58 to 9.86 μM) against the *L. donovani* strain originating from Ethiopia (experiment 1) and from 2.55 μg/mL to 4.06 μg/mL (corresponding to 6.26 to 9.96 μM) against the Indian *L. donovani* strain (experiment 2). Corresponding values for the respective reference standards were 4.99 μg/mL and 3.79 μg/mL for the Ethiopian strain and 5.92 μg/mL and 3.02 μg/mL for the Indian strain. In contrast no significant reduction in the number of infected macrophages was obtained with extracts from Miltefos™ capsules and no EC_50_ values could be determined. EC_50_ values are summarized in Table 2. The highest concentration of 100 μg/mL produced toxicity towards macrophages when exposed to API or extracts from Impavido^®^ capsules as judged by a reduction in the number of macrophages/well. At this concentration toxicity towards the host cell was expected for the API and extracts from capsules containing the stated amount of miltefosine. No toxicity was observed for cultures exposed to extracts from Miltefos™ capsules.

## Discussion

Drug quality issues have been raised previously over two types of the drugs currently used for the treatment of both VL and CL.^2^ The pentavalent antimonials have been used for the treatment of VL since 1937, CL since 1943, and for the mucocutaneous form of the disease since 1962. Despite this the chemical structure of the two commercially available forms, sodium stibogluconate (Pentostam^®^ or SSG) and *N*-Methylglucamine antimoniate (Glucantime^®^) have only recently been determined by Frezard and colleagues.^12^,^13^ Sodium stibogluconate appears as a complex mixture of polymeric forms, with batch-to-batch variation and solutions containing 32–34% pentavalent antimony (Sb^V^). Methylglucamine antimoniate has an Sb^V^ content that varies around 28% between batches. The major moieties of this oligomeric drug are Sb(v)—ligand complexes that are zwitterionic in solution. Treatment with antimonials is associated with a range of toxicities.^12^,^14^,^15^ Quality control in the face of this chemical complexity and batch-to-batch variation is essential. During a period when there were several manufacturers of these antimonial drugs, issues around drug quality and patient toxicity were reported.^16^ Studies by groups in India and Brazil reported significant differences in osmolarity and oxidation states between batches^17^,^18^ emphasising the need for standardization and monitoring product stability by the drug manufacturers.

In the case of miltefosine different analytical techniques of liquid chromatography coupled to MS-MS, Fourier transform infrared, and near-infrared spectroscopy have been previously used to characterize poor quality drugs and were used to determine the quality of generic Miltefos™ capsules collected in Bangladesh.^8^ We used direct injection MS to determine the molecular weight and fragmentation data to elucidate the structure, as well as NMR spectroscopy (^1^H and ^31^P) to characterize and definitively confirm the presence of atomic nuclei in a molecule. The molecule of miltefosine contains a phosphorous atom and proton atoms (Figure 1). Hence, the presence of a ^31^P peak on ^31^P NMR and proton splitting patterns on ^1^H NMR seen unequivocally confirm the authenticity of the Impavido^®^ capsules but not of Miltefos™. Our results based on determining the molecular structure of miltefosine, showed that the generic product is falsified (contained no API), whereas the quality assured product has the anticipated fragmentation patterns on MS and the peaks on both proton and phosphorous NMR analysis. Based on the NMR and MS spectra achieved the compound present in these falsified capsules may be palmitic acid. The excipients in the generic product have been reported to be lactose and microcrystalline cellulose (a typical excipient used in vitamins), and no stated API.^8^

Added to these results the bioassay analysis of extracts from Impavido^®^ capsules and the reference API displayed similar and expected anti-leishmanial activity with EC_50_ values below 5 μg/mL against intracellular *L. donovani* amastigotes in vitro. However, no significant anti-leishmanial activity was identified against two different strains of *L. donovani* by the contents from generic Miltefos™ capsules within the concentration ranges tested. No toxicity towards PEMs was observed with extracts from generic Miltefos™ capsules even at nominal miltefosine concentrations of up to 100 μg/mL.

These studies emphasize the role of drug regulators in ensuring the standards and quality of procured drugs.

## Conclusions

Substandard medicines (a formulation with too little or too much API) are a concern in countries with low standards of quality control of the manufacturing process (non-good manufacturing accredited status).^19^

The analytical chemical techniques of MS and NMR (proton and ^31^Phosphorous) confirmed that the generic Miltefos™ capsules (50 and 10 mg) did not contain the stated miltefosine and is classified as falsified (no stated API found). In contrast the analysis of the quality assured product Impavido^®^ (50 and 10 mg) from Aeterna Zentaris GmbH (Frankfurt, Germany) gave the anticipated ionisation patterns and spectra. The lack of API was confirmed with the absence of bioactivity of the generic product compared with the activity of the quality assured product. The arsenal of techniques made available to determine the quality of essential drugs will help to save lives and help the fight against counterfeiters. The ability to achieve confirmatory results using multiple techniques provides added reassurance to the validity of the findings.

The WHO Expert Committee on the control of leishmaniasis (2010) highlighted the importance of this fact emphasis to the governments to only acquire drugs from prequalified producers (see Annex at WHO TRS 949).^20^ The Bangladesh government removed Miltefos from the public health clinics (PHCs) and decided to follow WHO recommendations in using Ambisome^®^ (10 mg/kg total dose single infusion) as the first line treatment and is at present close to reaching the elimination goal of < 1 case in 10,000 in most of the upazillas after having treated more than 7,000 patients.^21^ Popular Laboratories was taken to court and told to stop the production of Miltefos™. However, they retained the right to import the API from China and were made to give assurance that they would produce a quality assured formulation of miltefosine.

National programs need quality assured drugs in their effort to eliminate diseases such as VL. Sensitive and specialized chemical techniques (NMR and MS) with experienced staff, are needed in bio-analytical laboratories in disease-endemic countries that can be used relatively quickly to determine the quality of drugs to treat fatal diseases.

## Figures and Tables

**Table 1:** Summary of results for the NMR and mass spectrometry analyses of the various miltefosine capsules*

Manufacturer	Stated dose, mg	Batch no.	Manufacturing date	Expiry date	API (miltefosine) detected or not by ^1^H, ^31^P NMR and MS
Impavido^®^	50	8J7717	07/2006	07/2012	Detected
Impavido^®^	10	8H7859	08/2008	08/2012	Detected
Miltefos™	50	SFD13 E0610	Not supplied	E0610	Not detected
Miltefos™	10	SFD12 E0610	Not supplied	E0610	Not detected

NMR = nuclear magnetic resonance; API = active pharmaceutical ingredient.

* The drugs were analyzed in October 2008 at the request of The World Health Organization (WHO).

**Table 2:** Anti-leishmanial activity of the reference API and capsule extracts*

Specification	Batch	Exp.	EC_50_ [μg/mL]
Reference standard 10 mg/mL	D-18506	1	4.99 (4.35–5.64)
Reference standard 2 mg/mL	D-18506	1	3.79 (3.74–3.83)
Impavido^®^ 50 mg miltefosine	8J7717	1	2.68 (1.15–4.21)
Impavido^®^ 10 mg miltefosine	8H7859	1	4.02 (2.65–5.39)
Miltefos™ 50 mg miltefosine	SFD13 E0610	1	> 100
Miltefos™ 10 mg miltefosine	SFD12 E0610	1	> 20
Reference standard 10 mg/mL	D-18506	2	5.92 (2.15–9.68)
Reference standard 2 mg/mL	D-18506	2	3.02 (1.81–4.24)
Impavido^®^ 50 mg miltefosine	8J7717	2	2.55 (1.41–3.69)
Impavido^®^ 10 mg miltefosine	8H7859	2	4.06 (2.63–5.48)
Miltefos^®^ 50 mg miltefosine	SFD13 E0610	2	> 100
Miltefos^®^ 10 mg miltefosine	SFD12 E0610	2	> 20

* EC_50_ values are given with 95% confidence intervals in brackets. The values for Miltefos™ are representative of two samples per batch and nominal drug concentration.

## References

[1] Alvar J, Vélez ID, Bern C, Herrero M, Desjeux P, Cano J, Jannin J, den Boer M (2012). Leishmaniasis worldwide and global estimates of its incidence. WHO Leishmaniasis Control Team. PLoS ONE.

[2] Croft SL, Olliaro P (2011). Leishmaniasis chemotherapy–challenges and opportunities. Clin Microbiol Infect.

[3] Sundar S, Jha TK, Thakur CP, Engel J, Sindermann H, Fischer C, Junge K, Bryceson A, Berman J (2002). Oral miltefosine for Indian visceral leishmaniasis. N Engl J Med.

[4] Bhattacharya SK, Sinha PK, Sundar S, Thakur CP, Jha TK, Pandey K, Das VR, Kumar N, Lal C, Verma N, Singh VP, Ranjan A, Verma RB, Anders G, Sindermann H, Ganguly NK (2007). Phase 4 trial of miltefosine for the treatment of Indian visceral leishmaniasis. J Infect Dis.

[5] Dorlo TP, Balasegaram M, Beijnen JH, de Vries PJ (2012). Miltefosine: a review of its pharmacology and therapeutic efficacy in the treatment of leishmaniasis. J Antimicrob Chemother.

[6] Sundar S, Mondal D, Rijal S, Bhattacharya S, Ghalib H, Kroeger A, Boelaert M, Desjeux P, Richter-Airijoki H, Harms G (2008). Implementation research to support the initiative on the elimination of kala azar from Bangladesh, India and Nepal–the challenges for diagnosis and treatment. Trop Med Int Health.

[7] Senior K (2008). Global health-care implications of substandard medicines. Lancet Infect Dis.

[8] Dorlo TP, Eggelte TA, de Vries PJ, Beijnen JH (2012). Characterization and identification of suspected counterfeit miltefosine capsules. Analyst (Lond).

[9] Dorlo TP, Hillebrand MJ, Rosing H, Eggelte TA, de Vries PJ, Beijnen JH (2008). Development and validation of a quantitative assay for the measurement of miltefosine in human plasma by liquid chromatography-tandem mass spectrometry. J Chromatogr B Analyt Technol Biomed Life Sci.

[10] Dorlo TP, Eggelte TA, Schoone GJ, de Dries PJ, Beijnen JH (2012). A poor-quality generic drug for the treatment of visceral leishmaniasis: a case report and appeal. PLoS Negl Trop Dis.

[11] Seifert K, Escobar P, Croft SL (2010). In vitro activity of anti-leishmanial drugs against *Leishmania donovani* is host cell dependent. J Antimicrob Chemother.

[12] Frezard F, Demicheli C, Ribeiro RR (2009). Pentavalent antimonials: new perspectives for old drugs. Molecules.

[13] Frézard F, Martins PS, Barbosa MC, Pimenta AM, Ferreira WA, de Melo JE, Mangrum JB, Demicheli C (2008). New insights into the chemical structure and composition of the pentavalent antimonial drugs, meglumine antimonate and sodium stibogluconate. J Inorg Biochem.

[14] Sundar S, Chakravarty J (2010). Antimony toxicity. Int J Environ Res Public Health.

[15] Marsden PD (1985). Pentavalent antimonials: old drugs for old diseases. Rev Soc Brasil Med Trop.

[16] Sundar S, Sinha PR, Agrawal NK, Srivastava R, Rainey PM, Berman JD, Murray HW, Singh VP (1998). A cluster of cases of severe cardiotoxicity among kala-azar patients treated with a high-osmolarity lot of sodium antimony gluconate. Am J Trop Med Hyg.

[17] Romero GA, de Oliveira MR, Corriera D, Marsden PD (1996). Physico-chemical characteristics of meglumine antimoniate in different conditions. Rev Soc Bras Med Trop.

[18] Franco MA, Barbosa AC, Rath S, Dorea JG (1995). Antimony oxidation states in antileishmanial drugs. Am J Trop Med Hyg.

[19] Caudron JM, Ford N, Henkens M, Mace C, Kiddle-Monroe R, Pinel J (2008). Substandard medicines in resource-poor settings: a problem that can no longer be ignored. Trop Med Int Health.

[20] World Health Organization (2010). Control of the Leishmaniases. Report of a meeting of the WHO Expert Committee on the Control of Leishmaniases, Geneva, March 22–26, 2010. WHO Technical Report Series.

[21] Chowdhury R, Mondal D, Chowdhury V, Faria S, Alvar J, Nabi SG, Boelaert M, Dash AP (2014). How far are we from visceral leishmaniasis elimination in Bangladesh? An assessment of epidemiological surveillance data. PLoS Negl Trop Dis.

